# Optimization of Ribosome Footprinting Conditions for Ribo-Seq in Human and *Drosophila melanogaster* Tissue Culture Cells

**DOI:** 10.3389/fmolb.2021.791455

**Published:** 2022-01-25

**Authors:** Katerina Douka, Michaela Agapiou, Isabel Birds, Julie L. Aspden

**Affiliations:** ^1^ School of Molecular and Cellular Biology, Faculty of Biological Sciences, University of Leeds, Leeds, United Kingdom; ^2^ LeedsOmics, University of Leeds, Leeds, United Kingdom

**Keywords:** Ribo-seq, ribosome profiling, footprinting, mRNA translation, ORF

## Abstract

Our understanding of mRNA translation and its regulation has been transformed by the development of ribosome profiling. This approach relies upon RNase footprinting of translating ribosomes in a precise manner to generate an accurate snapshot of ribosome positions with nucleotide resolution. Here we tested a variety of conditions, which contribute to the preciseness of ribosome footprinting and therefore the success of ribosome profiling. We found that NaCl concentration, RNaseI source, RNaseI amount, and temperature of footprinting all contributed to the quality of ribosome footprinting in human neuroblastoma SH-SY5Y cells. These ideal conditions for footprinting also improved footprint quality when used with *Drosophila melanogaster* S2 cells. Footprinting under the same conditions generated different footprints sizes and framing patterns in human and *D. melanogaster* cells. We also found that treatment of S2 cells with cycloheximide prior to footprinting impacted the distribution of footprints across ORFs, without affecting overall read length distribution and framing pattern, as previously found in other organisms. Together our results indicate that a variety of factors affect ribosome footprint quality and the nature of precise footprinting varies across species.

## Introduction

Next-generation sequencing approaches have transformed our understanding of gene expression and its regulation. RNA-seq based methods revolutionised the measurement of RNA species, levels, and splicing. However, mRNA translation lagged behind in its study at the genome-wide level until the development of ribosome profiling (Ribo-Seq) (Ingolia et al., 2009). The use of Ribo-Seq has transformed our understanding of the translatome, revealing translation of novel ORFs (Douka et al., 2021), stop-codon read through (Dunn et al., 2013), use of alternative initiation codons, (Van Damme et al., 2014) and providing mechanistic insights into translation elongation (Wu et al., 2019) and ribosome stalling (Rubio et al., 2021). This approach has been employed across a wide range of species and systems (Ingolia et al., 2009; Guo et al., 2010; Baltz et al., 2012; Ingolia et al., 2013; Aspden et al., 2014; Duncan and Mata, 2014; Chung et al., 2015; Heyer and Moore, 2016; Hsu et al., 2016). By isolating and sequencing the portion of RNA covered by the translational machinery, the ribosome, we can now perform transcriptome-wide assessments of protein translation and translational regulation.

Central to the success of ribosome profiling experiments is the ability to isolate ribosome footprints. The key experimental step in the generation of footprints is the RNase treatment of cytoplasmic lysate. Ribosome footprints are typically around 28–32 nucleotides (nt), although this varies by organism, cell or tissue type, and experimental protocol (Ingolia et al., 2013; Aspden et al., 2014). For Ribo-Seq data to be useful in the study of translation, the fragments of RNA sequenced need to originate from ribosome protected fragments rather than other RNA-protein complexes. Therefore, it is important to isolate RNA fragments corresponding to 28–32 nt ribosome footprints rather other smaller non-translation dependent fragments.

One of the important and distinctive features of Ribo-Seq data compared to RNA-seq data is its triplet periodicity. This is a bias in the mapping of reads toward one of the three possible reading frames, reflecting the codon-by-codon decoding activity of the ribosome. This pattern is not observed in RNA-seq, and a strong framing preference is indicative of high quality Ribo-Seq data. Triplet periodicity is assessed using metagene plots; aggregate plots which illustrate the distance between one end of the Ribo-Seq read (generally 5′) and the annotated start and stop codons of consensus coding sequence (CCDS). A clear preference for a single reading frame is expected. Metaplots can also be used to infer the position of the P-site of the ribosome with respect of the ribosome footprint read. This feature is important in determining precisely which codon is being decoded by each read. Together with framing, this information enables quality assessment of the ribosome profiling to be performed, and can also provide insight into the movement of the ribosome (Lareau et al., 2014; Wu et al., 2019).

The majority of standard Ribo-Seq reads are expected to map to canonical coding sequences, with a low percentage mapping to 5′-untranslated regions (UTRs) and very few to 3′-UTRs. Ribosome footprints mapping to 5′-UTRs generally represent scanning ribosomes, or the translation of upstream ORFs (uORFs) (Heyer & Moore, 2016; Rodriguez et al., 2019; Chen et al., 2020), while reads in the 3′-UTR may represent rarer downstream ORF (dORF) translation events. RNA-binding proteins (RBPs) can also create footprints, but these will not exhibit a framing bias, and may be of different lengths to true ribosome footprints (Ruiz-Orera and Albà, 2019). Although many technical improvements have been made to the original ribosome profiling protocol, there are several experimental variables that can be altered to optimise footprinting conditions. These include buffer conditions, temperature, amount and type of RNAseI. When working with a new organism or tissue type, or starting up ribosome profiling in a new lab, it is not always clear where to start, or how much variation from published data should be expected. Here we describe work to optimise footprinting conditions in human neuroblastoma SH-SY5Y cells and *Drosophila melanogaster* S2 cells. Together these experiments indicate key attributes which can affect the quality of ribosome profiling data in two different organisms although comparisons are only qualitative because additional replicates were not performed. Our results illustrate the impact experimental conditions can have on the final outputs of such experiments.

## Materials and Methods

### Cell Culture

Human neuroblastoma SH-SY5Y cells were cultured in Dulbecco’s Modified Eagle Medium (DMEM; 4.5 g/L Glucose with L-Glutamine) supplemented with 1% (v/v) Penicillin/Streptomycin (GE Healthcare) and 10% Fetal Bovine Serum (FBS, Sigma) at 37°C, 5% CO_2_. Semi-adherent *D. melanogaster* S2 cells were maintained in Schneider’s medium containing L-glutamine (Sigma) supplemented with 1% (v/v) Penicillin/Streptomycin/amphotericin B (GE Healthcare), 10% FBS, and maintained at 26°C in non-vented, adherent flasks (Sarstedt).

### Poly-Ribo-Seq

Cells were treated with cycloheximide (Sigma) at 100 μg/ml for 3 min at 37°C, washed (1X PBS, 100 μg/ml cycloheximide) and trypsinised for 5 min at 37°C. Subsequently, cells were pelleted, washed (1X PBS, 100 μg/ml cycloheximide), and resuspended in ice cold lysis buffer (Supplementary Table S1); 50 mM Tris-HCl pH8, 150 mM NaCl, 10mM MgCl_2_, 1 mM DTT, 1% IGEPAL, 100 μg/ml cycloheximide, Turbo DNase 24 U/mL (Invitrogen), RNasin Plus RNase Inhibitor 90 U (Promega), cOmplete Protease Inhibitor (Roche), for 45 min. Cells were then subjected to centrifugation at 17,000 × g for 5 min, to pellet nuclei. Cytoplasmic lysate was loaded onto 18–60% sucrose gradients (∼70 × 10^6^ cells per gradient) at 4°C and subjected to ultracentrifugation (121,355 × g_avg_ 3.5h, 4°C) in SW-40 rotor. Polysome fractions were pooled and diluted in either Buffer 1 (50 mM Tris-HCl pH8, 150 mM NaCl, 10 mM MgCl_2_) or Buffer 2 (100 mM Tris-HCl pH8, 30 mM NaCl, 10 mM MgCl_2_). RNaseI (either AM2295 at 10–20U/million cells, or EN601, 10 U/µl 0.7–1 U/million cells) was subsequently added incubated either for 1 h at RT or overnight at 4°C. RNaseI was deactivated using SUPERase inhibitor (200U/gradient) for 5 min at 4°C. Samples were concentrated using 30 kDa molecular weight cut-off columns (Merck) and loaded on sucrose cushion (1 M sucrose, 50 mM Tris-HCl pH8, 150 mM NaCl, 10mM MgCl_2_, 40U RNase Inhibitor) and subjected to ultracentrifugation at 204,428 × g_avg_ at 4°C for 4 h (TLA110). Pellets were resuspended in TRIzol (Ambion, Life Technologies) and processed for RNA purification followed by TURBO DNase treatment (Thermofisher) (according to manufacturer’s instructions), acidic phenol/chloroform RNA purification and ethanol precipitation at −80°C overnight. RNA concentration was determined by Nano-drop 2000 software. 28–34 nt ribosome footprints were gel purified in 10% (w/v) polyacrylamide-TBE-urea gel at 300 V for 3.5 h in 1X TBE. Ribosome footprints were subjected to rRNA depletion (Illumina, RiboZero rRNA removal kit).

### Ribo-Seq

Cycloheximide treated cells were treated for 3 min with 100 μg/ml cycloheximide before being pelleted. All cells were pelleted (8 min at 800 ×g), washed (1 × PBS, 100 μg/ml cycloheximide) and resuspended in ice cold lysis buffer and left to lyse for 45 min. Nuclei were removed via centrifugation (17,000 × g for 5 min) and cytoplasmic lysates were footprinted overnight at 4°C. Two different footprinting conditions were tested on both cycloheximide treated and untreated lysates: 1) A-RNAseI (AM2295) in Buffer 1 (50 mM Tris-HCl pH8, 150 mM NaCl, 10 mM MgCl_2_), 2) E-RNAseI (EN0601) in Buffer 2 (100 mM Tris-HCl pH8, 30 mM NaCl, 10 mM MgCl_2_). RNaseI was deactivated using SUPERase inhibitor (500°U/gradient) for 5 min at 4°C. Footprinted lysates were loaded onto sucrose gradients and subjected to ultracentrifugation at 4°C and subjected to ultracentrifugation (121,355 × g_avg_ 3.5h, 4°C) in SW-40 rotor. 80S ribosomes were purified away from ribosomal subunits and polysomes. RNA was isopropanol precipitated, TURBO DNase treated, acidic phenol/chloroform purified and ethanol precipitated at -80°C overnight. 28–34 nt ribosome footprints were gel purified via a 10% (w/v) polyacrylamide-TBE-urea gel (300°V, 3.5°h, 1X TBE), T4 PNK treated and isopropanol precipitated. rRNA depletion for S2 cells carried out with custom made beads. rRNA depleted footprints were ethanol precipitated again.

### Library Preparation and Sequencing

5′ stranded libraries were constructed using NEB Next Multiplex Small RNA Library Prep. Resulting cDNA was PCR amplified and gel purified prior to sequencing. Libraries were subjected to 75bp single end RNA Seq using NextSeq500 Illumina sequencer, High Output Kit v2.5 (75 Cycles) (Next Generation Sequencing Facility, Faculty of Medicine, University of Leeds).

### Ribosome Footprinting Analysis

Poly-Ribo-Seq and Ribo-Seq fastq files were uploaded on Ribogalaxy (Michel et al., 2016) and subjected to quality control using FastQC (v.0.11.5) (Andrews, 2010). 3′ end adapter sequence AGA​TCG​GAA​GAG​CAC​ACG​TCT was trimmed from the reads using Cutadapt (v.1.1) (Martin, 2011), discarding untrimmed footprint reads. Trimmed reads were further filtered, so that 90% of each read passed the quality threshold Phred score of 20, using the Filter by quality tool (Gordon, 2010) on Galaxy (Afgan et al., 2018). Subsequently, rRNA and tRNA reads were removed, using Bowtie (v.0.12.7) (Langmead and Salzberg, 2012) and 1 base trimmed from the 3′ end of reads. For assessment of the framing quality of ribosome footprinting, reads were mapped to the human (version hg38, Gencode v29) or the *D. melanogaster* (version dm3, BDGP Release 5) transcriptome and were subsequently processed with the RiboSeqR pipeline (Hardcastle, 2014). The analysis was performed on read lengths 25–35 nt, in order to assess the number of reads of each specific length that are in each frame. A metagene analysis was performed on the reads that display the best triplet periodicity (31 and 33 nt for human, 28 and 29 nt for fly) with parameters for filtering those reads (filterHits parameters) set as: lengths = 31, 33 (or 28, 29); frames = 1, 2, 3; hitMean = 50; unqhitMean = 10. Plots were generated and the plotCDS (parameters set as: lengths = 31, 33 (or 28, 29); min5p = -100; max5p = 100; min3p = −100; max3p = 100). In this analysis, reads were globally mapped to 5′ and 3′ UTRs and coding regions (CDS) and the mean number of reads that is mapped to each region is plotted.

### Translated ORF Detection

Quality reports of *D. melanogaster* Ribo-Seq and RNA-seq data were made using Fastqc (v.0.11.9) (Andrews, 2010). Adapter sequences (AGA​TCG​GAA​GAG​CAC​ACG​TCT) were trimmed using Cutadapt (v.2.10) (Martin, 2011) with minimum read length of 25bp, and untrimmed outputs retained for RNA-seq reads. Low-quality reads (score < 20 for 10% or more of read) were then discarded using FASTQ Quality Filter, FASTX-Toolkit (v.0.0.14) (Gordon, 2010). *D. melanogaster* rRNA sequences were retrieved from RiboGalaxy (Michel et al., 2016) and tRNA sequences from FlyBase release FB 2020_04 (Larkin et al., 2021). One base was removed from 3′ end of reads to improve alignment quality, and reads originating from rRNA and tRNA were aligned and removed using Bowtie2 (v.2.4.1) (Langmead and Salzberg, 2012).

The splice aware aligner STAR (v2.7.5c) (Dobin et al., 2012) was used to map remaining reads to the *D. melanogaster* reference genome (r6.35) from FlyBase (Larkin et al., 2021). The STAR (v2.7.5c) (Dobin et al., 2012) genome index was built with a sjdbOverhang of 99. Samtools (v.1.10) (Li et al., 2009) was used to create sorted, indexed bam files of the resulting alignments. These bam files were then subsampled to ∼2,000,000 reads per sample to create a fairer comparison. Alignments were visualised using Golden Helix GenomeBrowse (v3.0.0).

Metaplots of aligned Ribo-Seq data were generated using create_metaplots.bash script from Ribotaper (v1.3) pipeline (Calviello et al., 2016). These show the distance between the 5′ ends of Ribo-Seq and annotated start and stop codons from CCDS ORFs, allowing the locations of P-sites to be inferred. Read lengths exhibiting the best triplet periodicity were selected for each replicate, along with appropriate offsets (Supplementary Table S2).

Translated smORFs were then identified using Ribotaper (v1.3) (Calviello et al., 2016). Initially, this requires an exon to contain more than 5 P-sites in order to pass to quality control steps. Identified ORFs were then required to have a 3-nt periodic pattern of Ribo-Seq reads, with 50% or more of the P-sites in-frame. In the case of multiple start codons, the most upstream in-frame start codon with a minimum of 5 P-sites in between it and the next ATG was selected. ORFs for which >30% of the Ribo-Seq coverage was only supported by multimapping reads were also subsequently filtered (Chothani et al., 2019).

### General Statistics and Plots

Statistical analyses were performed in R (R Core Team, 2020) using packages including stringr (Wickham, 2019), dplyr (Wickham et al., 2017), ggplot2 (Wickham, 2016), knitr (Xie, 2020), eulerr (Larsson, 2020), viridis (Garnier et al., 2021) and tidyverse (Wickham et al., 2019).

## Results

### Changes to RNaseI Footprinting Affects Size and Framing of Ribosome Footprints in Human SH-SY5Y Cells

The precise conditions in which ribosome footprinting is performed can have substantial impact on the quality of the ribosome profiling experiment, as judged by the preciseness of the footprint and the level of triplet periodicity (framing). Although additional attributes can be used to assess the reproducibility of transcript-specific ribosome occupancy, we have focused on footprint size and triplet periodicity to specifically assess the quality of ribosome footprinting rather than other aspects of ribosome profiling. Triplet periodicity is particularly important when attempting to identify novel ORFs to ensure footprints represent elongating ribosomes rather than non-specific protein or ribosome binding. We previously developed an adaptation to Ribo-Seq, Poly-Ribo-Seq in *D. melanogaster* S2 cells (Aspden et al., 2014) (Supplementary Figure S1). By ribosome footprinting polysomal complexes rather than all ribosomal complexes, i.e., monosomes and polysomes, Poly-Ribo-Seq aids detection of genuine translation events in small or noncanonical ORFs.

**FIGURE 1:**
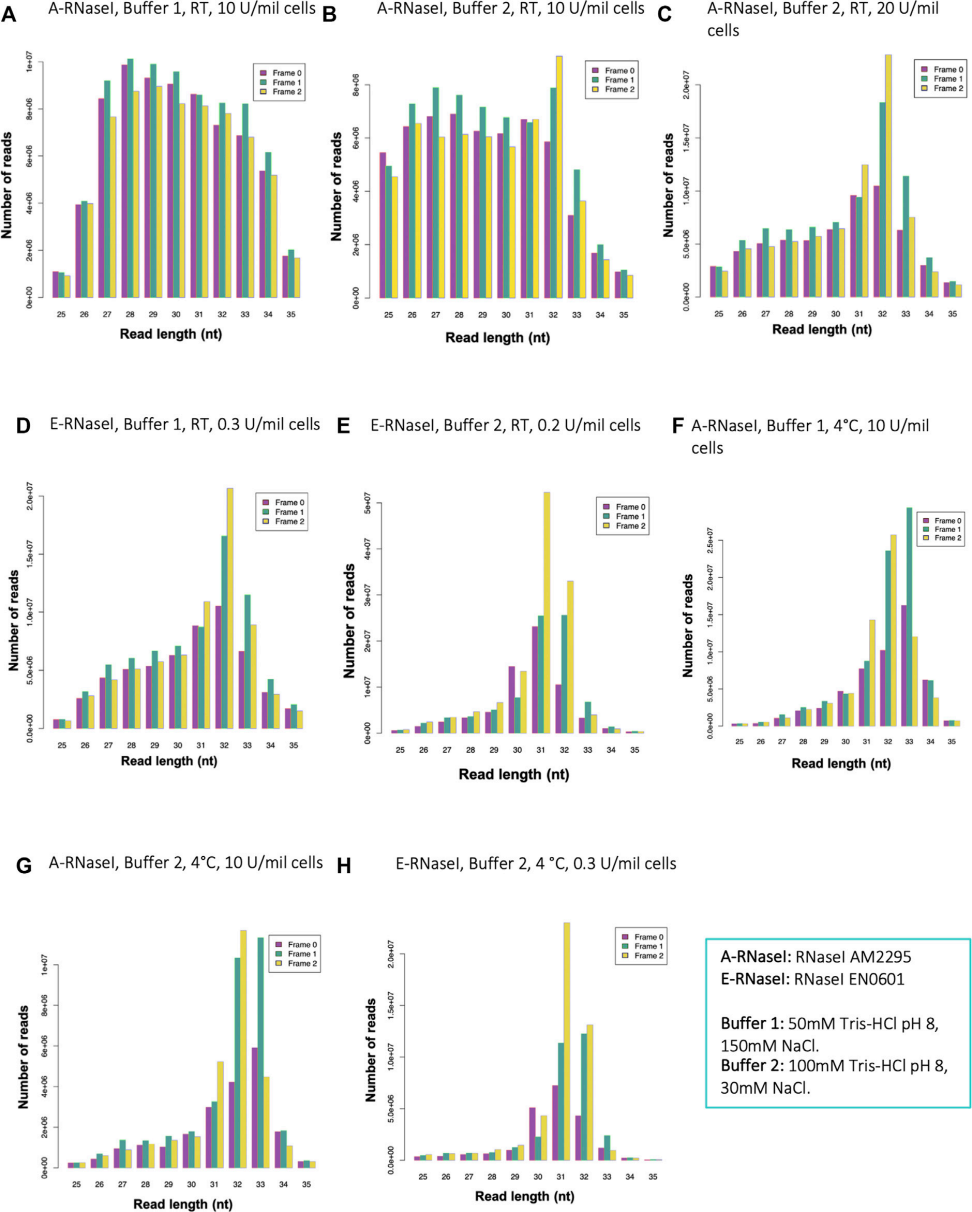
Changes to RNaseI footprinting affects size and framing of ribosome footprints in human SH-SY5Y cells. Read length distribution and frame plots, generated by RiboSeqR, from ribosome footprinting in cells SH-SY5Y under different conditions: **(A)** Buffer 1 (50 mM Tris-HCl pH8, 150 mM NaCl), RT 1 h, with A-RNaseI 10 U/million cells, **(B)** Buffer 2 (100 mM Tris-HCl pH8, 30 mM NaCl), RT 1 h, with A-RNaseI 10 U/million cells, **(C)** Buffer 2, RT 1 h, with A-RNaseI 20 U/million cells, **(D)** Buffer 1, RT 1 h, with E-RNaseI 0.3 U/million cells, **(E)** Buffer 2, RT 1 h, with E-RNaseI 0.2 U/million cells, **(F)** Buffer 1, O/N at 4°C, with A-RNaseI 10 U/million cells, **(G)** Buffer 2, O/N at 4°C, with A-RNaseI 10 U/million cells, **(H)** Buffer 2, O/N at 4°C, with E-RNaseI 0.3 U/million cells. See Table 1 for full details of each condition tested.

To employ Poly-Ribo-Seq for the first time in human neuroblastoma SH-SY5Y cells, and identify novel translation events, we initially tried the same footprinting conditions previously performed in *D. melanogaster* S2 cells. This included 50 mM Tris-HCl pH8, 150 mM NaCl, 10 mM MgCl_2_ and RNase I (AM2295) at 10 U/million cells, but with footprinting performed at room temperature (RT) for 1 h, as is standard for human cells (McGlincy, 2017; Wu et al., 2019). On the urea-acrylamide gel used to purify footprints, a smeary band corresponding to ribosome footprints was visible between the RNA markers of 28 and 34 nt (Supplementary Figure S2A). However, ribosome footprinting under these conditions resulted in ribosome footprint reads with a wide length distribution and virtually no triplet periodicity (Figure 1A). Most footprints did map to CCDSs indicating that they represented ribosomes (Supplementary Figure S3A), but with some noise within 5′-UTRs and imprecisely footprinted. Therefore, we sought to test a range of factors to improve the preciseness of the ribosome footprinting. Given these experiments were simply testing conditions only single replicates were performed.

Previously others have found in *Arabidopsis thaliana* that the buffer conditions of the footprinting buffer can affect RNAseI activity and therefore footprinting (Hsu et al., 2016). To emulate these conditions, we modified the buffer to reduce NaCl from 150 to 30 mM, and increased Tris-HCl pH8 from 50 to 100 mM, to test if this improved footprinting. We refer to the 50 mM Tris-HCl pH8, 150 mM NaCl buffer as Buffer 1, and the 100 mM Tris-HCl pH8, 30 mM NaCl buffer as Buffer 2. These changes to the buffer conditions did have a small effect on improving triplet periodicity of reads (Figure 1B). However, reads still showed a wide length distribution and low triplet periodicity. Therefore, we modified aspects of the RNase treatment to try and improve quality of footprinting. Increasing the amount of RNaseI present in the footprinting reaction dramatically improved both length distribution and framing (Figure 1C). The ratio of the amount of RNaseI enzyme to the RNA present in the reaction is important to ensure precise footprinting. Therefore the amount of RNaseI used in each experiment was adjusted based on the number of cells being subjected to footprinting, to maintain a consistent ratio between RNaseI and RNA.

Several Ribo-Seq publications have used alternative sources of RNaseI (McGlincy, 2017). Therefore, we tested EN0601 RNase (Thermofisher), E-RNaseI, alongside the AM2295 (Ambion), A-RNaseI, we previously used (Table 1). This E-RNaseI, when used in Buffer 1, also led to an improvement in triplet periodicity and footprint length distribution (Figure 1D). A combination of E-RNaseI and Buffer 2, together resulted in a substantial improvement to preciseness of footprinting (Figure 1E). Under these conditions 72% of footprints were 31–32 nt in length. The most abundant read length (31 nt) exhibits high levels of framing with 52% of 31 nt read in frame 2 (Supplementary Figure S3B).

**TABLE 1:** Summary of ribosome footprinting conditions tested with human SH-SY5Y cells.

Panel in Figure 2	RNaseI	Tris-HCl and NaCl concentrations	Footprinting temperature	Footprinting time
A	AM2295 10 U/million cells	50 mM Tris-HCl pH8, 150 mM NaCl (Buffer 1)	RT	1 h
B	AM2295 10 U/million cells	100 mM Tris-HCl pH8, 30 mM NaCl (Buffer 2)	RT	1 h
C	AM2295 20 U/million cells	100 mM Tris-HCl pH8, 30 mM NaCl (Buffer 2)	RT	1 h
D	EN0601 0.3 U/million cells	50 mM Tris-HCl pH8, 150 mM NaCl (Buffer 1)	RT	1 h
E	EN0601 0.2 U/million cells	100 mM Tris-HCl pH8, 30 mM NaCl (Buffer 2)	RT	1 h
F	AM2295 10 U/million cells	50 mM Tris-HCl pH8, 150 mM NaCl (Buffer 1)	4°C	overnight
G	AM2295 10 U/million cells	100 mM Tris-HCl pH8, 30 mM NaCl (Buffer 2)	4°C	overnight
H	EN0601 0.3 U/million cells	100 mM Tris-HCl pH8, 30 mM NaCl (Buffer 2)	4°C	overnight

Ribosome footprinting conditions tested in human SH-SY5Y cells with reference to data in Figure 1, RNaseI type and units, Tris-HCl pH8 and NaCl concentrations, incubation temperature and incubation time.

Previously in *D. melanogaster* we had performed footprinting at 4°C overnight to maintain stable ribosomes. However, the majority of Ribo-Seq experiments in human cells are performed at RT for 1 h (McGlincy, 2017; Wu et al., 2019). Performing footprinting at 4°C overnight with the A-RNaseI in Buffer 1, resulted in footprints with precise footprint of 31–33 nt, with good framing (Figure 1F, compared to Figure 1A). The temperature of footprinting clearly contributes a substantial improvement. An almost identical pattern was found when performed with Buffer 2 (Figure 1G), suggesting that the buffer has less of an effect on footprinting when performed at 4°C overnight compared to at RT for 1 h. In attempt to maximise the number of ribosomes remaining intact as 80S ribosomes bound to mRNAs, samples were also loaded onto gradients at 4°C, as well as footprinted at 4°C. This combination had little effect in the context of E-RNaseI (Figure 1H compared to Figure 1E).

Together the ribosome profiling conditions tested (Table 1) indicate that a number of factors contribute to the effectiveness of ribosome footprinting (Table 2). Buffer conditions can be modified to improve quality of footprints but in general it was more straightforward to achieve high quality footprints with E-RNaseI. Reducing the temperature, changing the buffer, and increasing amount of RNaseI all helped improved quality. For our cells of interest, human SH-SY5Y cells, we identified the best conditions (of those we tested) to be E-RNAaseI, 100 mM Tris-HCl pH8, 30 mM NaCl, ON at 4°C (Figure 1H), which produced the highest level of periodicity (Supplementary Figures S3C,D). Comparing metagene plots from these ‘best’ conditions, with those we started with (A-RNAaseI, 50 mM Tris-HCl pH8, 150 mM NaCl, RT for 1 h), at their ideal read lengths, background signal has been reduced substantially. Specifically, there are fewer reads mapping to UTRs in these improved conditions (Supplementary Figures S3D,E) compared to starting conditions (Supplementary Figure S3A).

**TABLE 2:** Summary conclusions from conditions tested.

Test	Background	Panels	Conclusion	Triplet periodicty (%)	Read length
Buffer conditions	A-RNAseI RT for 1 h	A and B	100 mM Tris-HCl, 30 mM NaCl > 50 mM Tris-HCl, 150 mM NaCl	2	Little difference
Buffer conditions	E-RNaseI RT for 1 h	D and E	100 mM Tris-HCl, 30 mM NaCl > 50 mM Tris-HCl, 150 mM NaCl	9	Increase in % of 31-32 nt reads
Buffer conditions	A-RNaseI, 4°C ON	F and G	100 mM Tris-HCl, 30 mM NaCl = 50 mM Tris-HCl, 150 mM NaCl	1	No difference. Both high % of 31-33 nt reads
RNaseI quantity	100 mM Tris-HCl pH8, 30 mM NaCl, A-RNAseI RT for 1 h	B and C	20U/million cells >> 10U/million cells	4	Large increase in % of 31-33 nt reads
RNaseI source	50 mM Tris-HCl pH8, 150 mM NaCl, RT for 1 h	A and D	E-RNaseI >>> A-RNaseI	6	Large increase in % of 31-33 nt reads
RNaseI source	100 mM Tris-HCl pH8, 30 mM NaCl, 4°C ON	G and H	E-RNaseI = A-RNaseI	3	Shift from 32 to 33 nt to 31–32 nt reads
RNaseI source	100 mM Tris-HCl pH8, 30 mM NaCl, RT for 1 h	C and E	E-RNaseI >> A-RNaseI	8	Moderate increase in % of 31-32 nt reads. Shift from 31 to 33 nt to 30–32 nt
Temperature	A-RNAseI, 50 mM Tris-HCl pH8, 150 mM NaCl,	A and F	4°C ON >>> RT for 1 h	13	Large increase in % of 31-33 nt reads
Temperature	E-RNAseI, 100 mM Tris-HCl pH8, 30 mM NaCl,	E and H	4°C ON = RT for 1 h	3	No difference in % of 31-32 nt reads
Temperature	E-RNAseI, 100 mM Tris-HCl pH8, 30 mM NaCl,	B and G	4°C ON >>> RT for 1 h	12	Large increase in % of 31-33 nt reads

Details of different tests performed and in what background conditions, which panels in Figure 1 show the results and conclusion of which condition achieved better footprint length and framing. Measures of changes in triplet periodicity (% difference in dominant frame for read length with best framing) and read length distribution (difference in % read length distribution and read length with best periodicity).

### Changes to RNaseI Footprinting Affects Size and Framing of Ribosome Footprints in *Drosophila* S2 Cells

**FIGURE 2:**
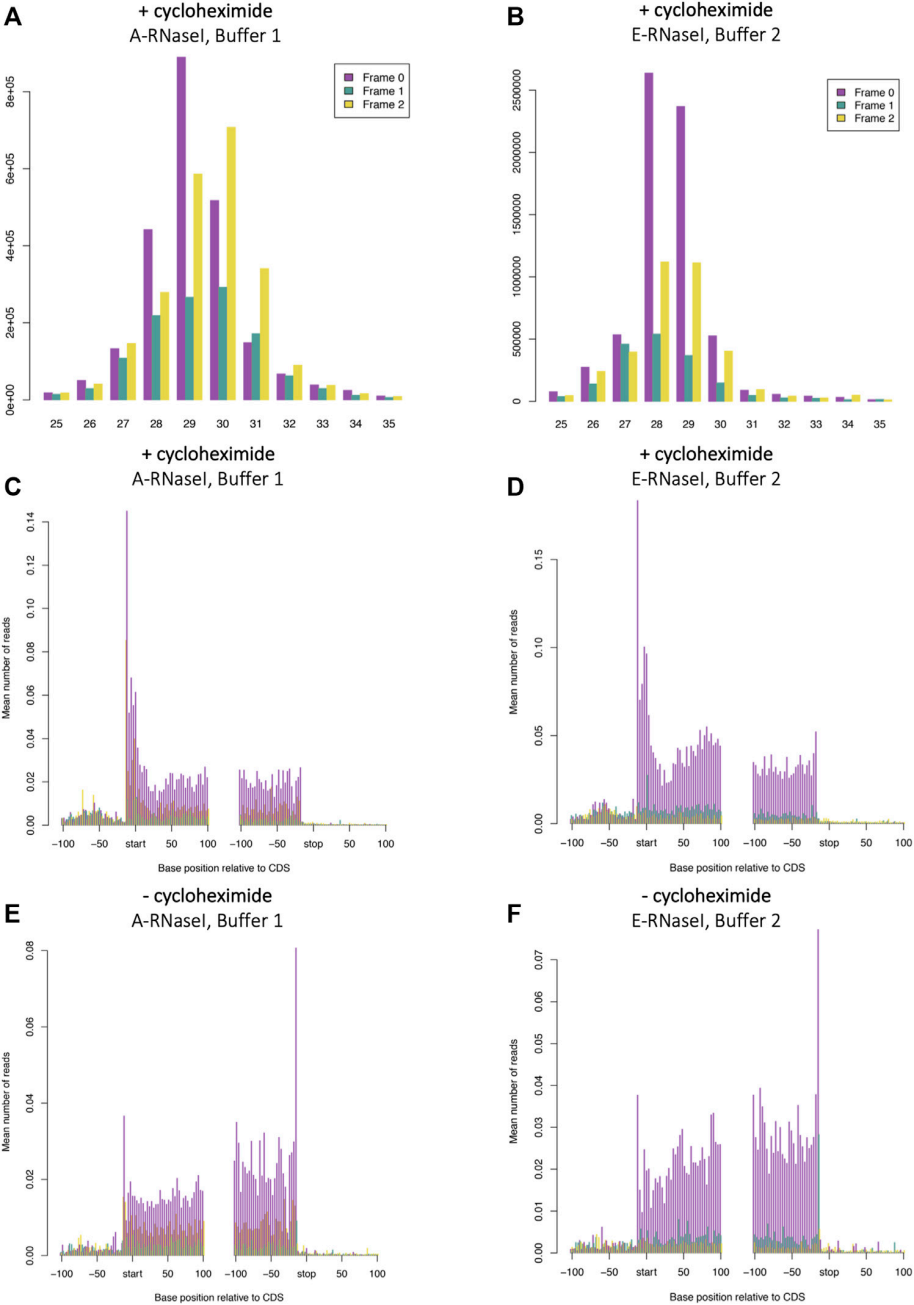
Changes to RNaseI footprinting affects size and framing of ribosome footprints in *Drosophila* S2 cells. Read length distribution and frame plots from Ribosome footprinting in *D. melanogaster* S2 cells in **(A)** A-RNaseI and Buffer 1 (50 mM Tris-HCl pH8, 150 mM NaCl) and **(B)** E-RNaseI and Buffer 2 (100 mM Tris-HCl pH8, 30 mM NaCl) footprinting conditions, both in presence of cycloheximide (100 μg/ml). Metagene plots from 29 nt ribosome footprints **(C and E)** A-RNaseI and Buffer 1 and metagene plots from 28 nt ribosome footprints **(D and F)** E-RNaseI and Buffer 2 footprinting conditions, either in **(C and D)** the presence or **(E and F)** absence of cycloheximide. Plots were generated with RiboSeqR.

To determine whether the improvements tested in human SH-SY5Y cells would also affect footprinting in *D. melanogaster* we performed Ribo-Seq on S2 cells in the best conditions we identified in SH-SY5Y cells (Buffer 2: 100 mM Tris-HCl pH8, 30 mM NaCl and ∼0.4 U/million cells E-RNaseI). Bands corresponding to ribosome footprints were visible on urea-acrylamide gels between 28 and 34 nt RNA markers (Supplementary Figure S2B). When compared with the ‘old’ conditions (Buffer 1: 50 mM Tris-HCl pH 8 and 150 mM NaCl and ∼20 U/million cells A-RNaseI, these new conditions made a substantial improvement to effectiveness of footprinting in *D. melanogaster* S2 cells (compare Figures 2A,B). The majority of footprints are 28–29 nt under these Buffer 2 and E-RNaseI conditions, compared to 28–31 nt in Buffer 1 with A-RNaseI conditions (Figures 2A,B). The proportion of reads exhibiting triplet periodicity is improved substantially from 51% of 29 nt reads in frame 0 (Figure 2A) to 61% of 28 nt reads in frame 0 (Figure 2B). The improvements to footprint length distribution and triplet periodicity were seen both in the presence (Figures 2A,B), absence of cycloheximide (Supplementary Figures S4A,B). Both footprinting conditions tested in S2 cells resulted in the majority of footprints mapping to coding sequences (CDSs), as evident in metagene analysis (Figures 2C,D).

Downstream analysis of the Ribo-Seq data revealed that the improved footprinting conditions identified more actively translated ORFs, both in the presence and absence of cycloheximide (Table 3). The number of CCDS ORFs - ORFs which overlap known coding regions in CCDS genes - increased by ∼1/5 in the new conditions (from 7,511 to 9,654), while the number of upstream ORFs (uORFs) detected nearly doubled (from 39 to 71). This indicates that the improved ribosome footprinting conditions not only increase triplet periodicity and preciseness of footprints, but also lead to the better detection of translation.

**TABLE 3:** Summary of translated ORFs identifed in *Drosophila* S2 cells.

ORF type	**A-RNaseI, Buffer 1 (+cycloheximide)**	**E-RNaseI, Buffer 2 (+cycloheximide)**	*A-RNaseI, Buffer 1 (- cycloheximide)*	*E-RNaseI, Buffer 2 (-cycloheximide)*
dORFs	**1**	**3**	*1*	*2*
ncORFS	**5**	**4**	*2*	*1*
CCDS ORFs	**7511**	**9654**	*5957*	*8407*
uORFs	**39**	**71**	*7*	*23*

Translated ORFs identified from ribosome profiling in *D. melanogaster* S2 cells in A-RNaseI, Buffer 1 and E-RNaseI, Buffer 2, footprinting conditions, in the presence (bold) or absence of cycloheximide (italic). The E-RNaseI, Buffer 2, conditions, which produce better quality framing find more ORFs both with and without cycloheximide. ORF types include downstream ORFs (dORFs) found downstream of the main ORF, non-coding ORFs (ncORFs) found on transcripts currently annotated as non-coding, CCDS ORFs overlap known coding regions in CCDS genes, and upstream ORFs (uORFs) are found upstream of the main ORF.

### Cycloheximide Treatment Affects Ribosome Footprint Distribution and Length

Previous Poly-Ribo-Seq in *D. melanogaster* S2 cells had only achieved modest framing and was performed in the presence of cycloheximide (Aspden et al., 2014). Therefore, we sought to determine the effect the addition of cycloheximide has in *D. melanogaster*. This is of particular interest for dissected tissues from numerous individual organisms (e.g. *D. melanogaster* testes) because batch flash freezing is not straightforward, and therefore cycloheximide is likely useful to trap elongating ribosomes.

**FIGURE 3:**
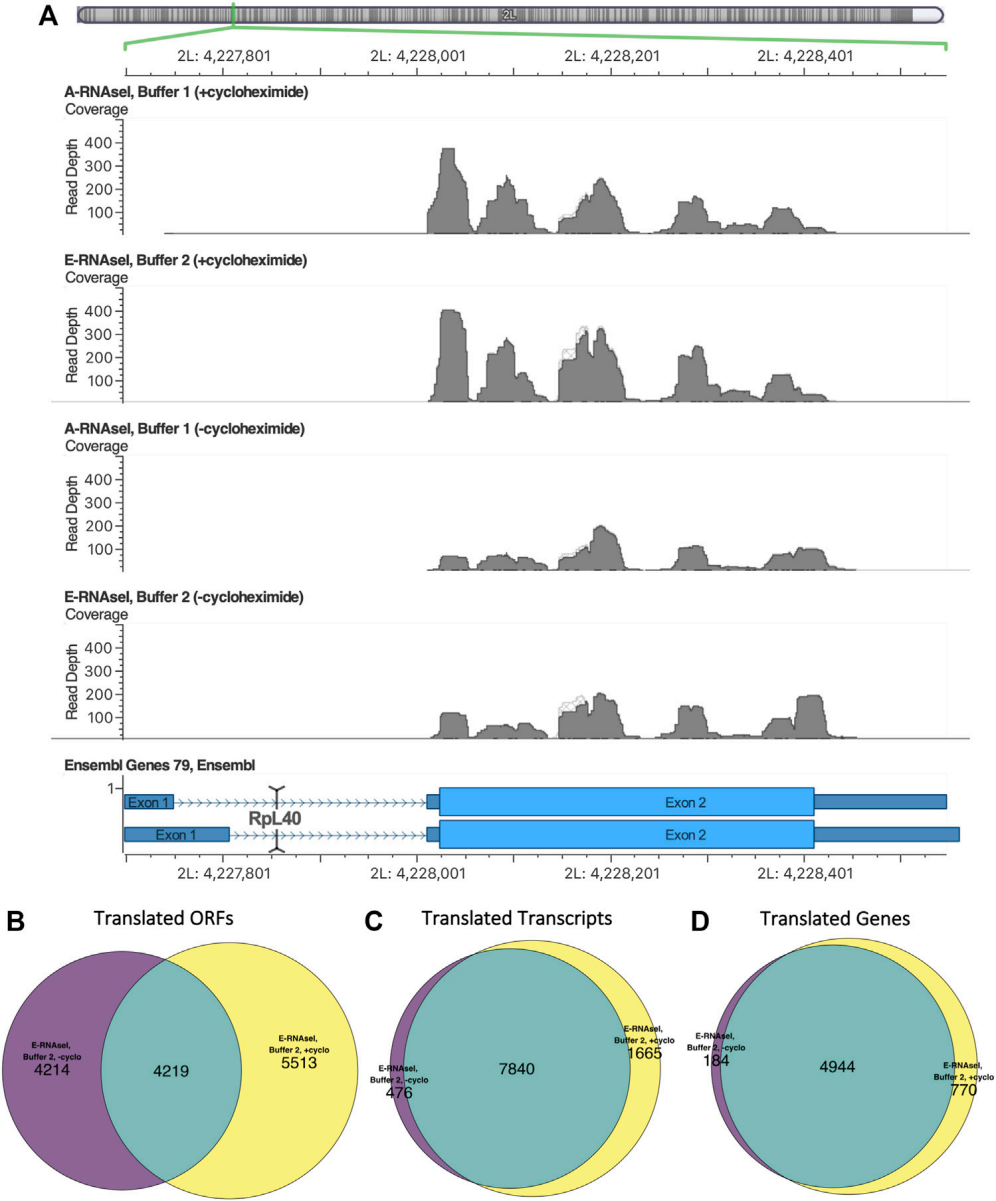
Differences in footprinting in the presence and absence of cycloheximide in *Drosophila* S2 cells. **(A)** Ribo-seq reads mapping to RpL40 in *D. melanogaster* S2 cells viewed using Golden Helix GenomeBrowse (v3.0.0). The presence/absence of cycloheximide causes changes in footprint build up. Replicates with cycloheximide exhibit build up at the start of the ORF, and replicates without cycloheximide have a build up at the end of the ORF, reflecting a ribosomal rearrangement at the stop codon. The overlap in translation events identified in the presence and absence of cycloheximide at the **(B)** ORF, **(C)** transcript and **(D)** gene level in *D. melanogaster* S2 cells, footprinted with E-RNaseI in Buffer 2.

To assess the effect of cycloheximide treatment Ribo-Seq was performed with S2 cells in the presence or absence of cycloheximide (final 100 µM), in the footprinting conditions used previously in *D. melanogaster* S2 cells (Aspden et al., 2014). These were 50 mM Tris-HCl pH8, 150 mM NaCl, 10 mM MgCl_2_ (i.e. Buffer 1) and A-RNaseI (∼20U/million cells). Metagene analysis revealed that cycloheximide treatment had a limited effect on footprint length or periodicity with Buffer 1, A-RNaseI (comparing Supplementary Figure S4A and Figure 3A) and Buffer 2 E-RNAseI (comparing Supplementary Figure S4B and Figure 2B). The distribution of reads across CDSs is affected by cycloheximide treatment, as previously described in other organisms (Duncan and Mata, 2017; Gerashchenko and Gladyshev, 2014; Hussmann et al., 2015; Sharma et al., 2019). Specifically, cycloheximide treatment results in a build-up of Ribo-Seq reads at the start codon and in the first ∼15 nt of CDSs (Figure 2C). Whilst in the absence of cycloheximide there is a build up around the stop codon (Figure 2E). These footprints around the stop codon are of a different frame compared within the main part of the CDS (frame 1 rather than 0), reflecting a ribosomal rearrangement at the stop codon (Lareau et al., 2014; Wu et al., 2019). This same pattern of effect by cycloheximide can also be seen in the improved conditions that used E-RNaseI and Buffer 2 by metagene analysis (comparing Figure 2D and Figure 2F), footprint length and framing (comparing Supplementary Figure S4B and Figure 2B).

The changes of Ribo-Seq read build up caused by cycloheximide can also be seen at the transcript level (Figure 3A). Ribosomal protein L40 (RpL40) was found to be translated (from transcript FBtr0334787) in all conditions. In samples treated with cycloheximide there is a build-up of reads at the start of the ORF, whereas in untreated samples we can see a pile up at the 3′ end of the ORF. The effects of cycloheximide on global changes to footprinting caused by cycloheximide can also be observed, with more ORFs detected in presence of cycloheximide (Table 3). At the ORF level, only ∼50% of the translated ORFs detected (in Buffer 2, E-RNase) in the absence of cycloheximide were also identified in the presence of cycloheximide (Figure 3B). At the transcript level, 94% of the transcripts were detected as translated in the absence of cycloheximide were also translated in presence of cycloheximide (Figure 3C), and 96% at the gene level (Figure 3D). Although this analysis is based on single samples, biological replicates would likely increase this overlap. This indicates that although the same translation events are likely to be taking place in both presence and absence of cycloheximide, the exact ORF a translation event is attributed to can be affected by the accumulation of reads at the start codon upon cycloheximide treatment.

### Length of Footprints and Nature of Framing is Different Between Humans and *Drosophila*

**FIGURE 4:**
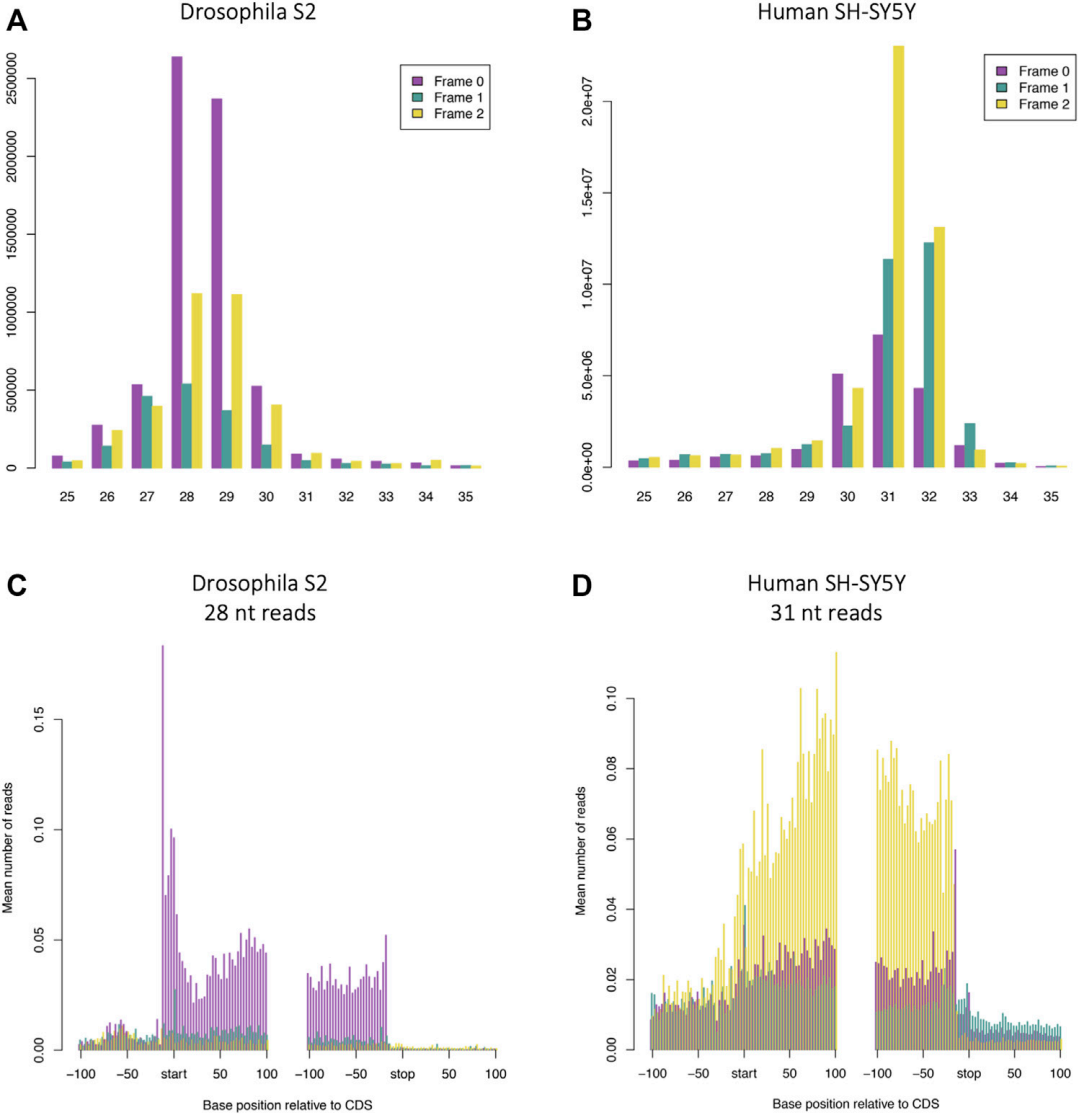
Length of footprints and nature of framing is different between Poly-Ribo-Seq in humans and Ribo-Seq in *Drosophila*. Read length distribution and framing of ribosome footprinting **(A)** *Drosophila* S2 cells **(**same as Figure 2B **)** and **(B)** human neuroblastoma SH-SY5Y cells **(**same as Figure 1G **)**, with E-RNaseI, in Buffer 2 at ON at 4°C, in presence of cycloheximide. Metagene analysis of ribosome profiling in **(C)** *Drosophila* S2 cells: 28 nt reads **(**same as Figure 2D **)** and **(D)** human neuroblastoma SH-SY5Y cells 31 nt reads **(**same as Supplementary Figure S3D **)**. Plots generated with RiboSeqR.

One of the most time-consuming aspects of performing Ribo-Seq is the requirement to find ideal conditions for footprinting. An added complication is that comparing your data to published data sets can indicate that there may be a problem with your own footprinting, but this may represent an actual difference in footprint length and pattern of framing between different systems. Here we have performed Poly-Ribo-Seq on human SH-SY5Y cells and Ribo-Seq on *D. melanogaster* S2 cells under the same conditions: 100 mM Tris-HCl pH8, 30 mM NaCl, 10 mM MgCl_2_ (Buffer 2) and E-RNaseI (∼0.4 U/million cells). This allows us to make direct comparisons of differences between the two. Under these conditions the majority (57.1%) of *D. melanogaster* S2 cells footprints are 28–29 nt in length (Figure 4A), whilst in human SH-SY5Y cells they are longer: 31–32 nt (Figure 4B). The pattern of triplet periodicity is also different with Frame 0 the dominant frame in S2 cells and Frame 2 in SH-SY5Y cells (Figures 4A,B). There are also differences in the metagene profiles, with S2 cells exhibiting a higher peak of reads around the start codon and stop codon (in presence of cycloheximide) (Figure 4C) when compared with human SH-SY5Y cells (Figure 4D) (also in the presence of cycloheximide). Signal in 5′-UTRs and 3′-UTRs is higher in Poly-Ribo-Seq of human SH-SY5Y cells (Figure 4D) compared with Ribo-Seq of S2 cells (Figure 4C). This may have more to do with the different sucrose gradients used in Poly-Ribo-Seq compared to Ribo-Seq, but we cannot be sure. Comparing similar footprinting conditions between different organisms and systems can generate subtle differences in footprinting nature, but as long as footprints display substantial framing and precise length distribution, translation can be detected and measured.

## Discussion

In this work we have tested a variety of experimental conditions which affect the quality of ribosome footprinting in human neuroblastoma SH-SY5Y cells and *D. melanogaster* S2 cells. Since no replicates were performed, the comparisons we report are only qualitative but our results could be beneficial to those performing ribosome profiling and adapting to new systems. NaCl and Tris-HCl concentrations, RNaseI source, RNaseI amount, and temperature of footprinting all contributed to the quality of ribosome footprinting. This highlights some key contributing factors to the success of ribosome footprinting that may not be obvious to the beginner. Although many standard ribosome profiling protocols perform ribosome footprinting at RT, others have also found that reducing the temperature to 4°C can reduce ribosome sensitivity to RNaseI in human cell lines (Cenik et al., 2015). The amount of nuclease has also been shown to affect footprinting efficiency in other systems (Dunn et al., 2013) and small quantities of ribosomes are particularly sensitive to the amount of RNaseI during footprinting (Liu et al., 2019). We, like others, have found it important to optimize ribosome footprinting conditions for the type of material and RNase that is being used. Not all RNases respond in the same way to changes in the other conditions, as we found for the two RNaseI we tested. An additional consideration is the wide variation in activity between *E. coli* RNAseI enzymes, that use different unit definitions to measure the enzyme activity (McGlincy, 2017; Liu et al., 2019) and the need to adjust for this, as well as potential variation between RNase batches. Our results were generated from single batches of both RNaseI sources. The type of RNase has also been previously shown to impact ribosome footprinting. For example, *Drosophila* ribosomes have been shown to be sensitive to digestion of their rRNA by RNaseI at higher temperatures (e.g. RT) so alternative RNases have been employed such as micrococcal nuclease (Dunn et al., 2013). The disadvantage of several alternatives to RNaseI, such as micrococcal nuclease, RNaseA and RNaseT1, is weaker triplet periodicity (Gerashchenko and Gladyshev, 2017). Several labs are now also using combinations of RNases for footprinting to reduce bias, minimise degradation of ribosomes and maximise triplet periodicity (Liu et al., 2019).

By comparing the optimisation of the ribosome footprinting in these disparate organisms, we demonstrate that footprinting under the same conditions can generate different footprint sizes and framing patterns. We therefore recommend you use existing literature and consult experts to plan experimental conditions and establish a reasonable range of expected footprint lengths when working with a new species. Even within the same species, variation should be expected when working with a different cell or tissue type. A key consideration for undertaking optimisation such as we describe is the balance between time and money spent, and the resulting improvement in footprinting quality. If one is establishing a protocol to support multiple studies in the same model and multiple replicates, this step is worth sustained investment.

An important consideration for ribosome profiling is the ‘trapping’ of ribosomes in the act of translation to provide an accurate snapshot of translation. Many researchers have relied upon cycloheximide treatment to aid this stabilisation of 80S ribosomes on the mRNA. However, as others have previously shown in yeast (Duncan and Mata, 2017), we found that cycloheximide can affect read distribution and ORF detection in *Drosophila* cells. However, it seems likely that cycloheximide treatment has less of an effect in humans and some other organisms, compared to yeast and fly, not impacting transcript-specific ribosome occupancy (Sharma et al., 2021). Both the results presented here in *Drosophila* and other studies have shown that cycloheximide treatment does not affect either footprint size distribution or framing (Sharma et al., 2019). Snap freezing material is an alternative to ‘trapping’ ribosomes during elongation using cycloheximde, which does not seem to affect framing, read length or distribution. Flash freezing can also be of benefit when collecting difficult or biologically challenging tissues. But there are circumstances where collecting tissues from individual animals over long time frames when cycloheximide treatment is logistically more appropriate. Overall, this study shows the importance of testing ribosome footprinting conditions in a new system and in combination different conditions can vary in their contribution to generating high quality ribosome profiling data.

## Data Availability

*D. melanogaster* S2 cell Ribo-seq data is deposited in GEO, record GSE166408. Human SH-SY5Y Ribo-seq data is deposited in SRA, BioProject PRJNA753469. Previously published *D. melanogaster* RNA-seq is available from GEO record GSE60384, run SRR1548661 (Aspden et al. 2014).
