# Supplementary material for: Optimization of Ribosome Footprinting Conditions for Ribo-Seq in Human and Drosophila melanogaster Tissue Culture Cells
Source: Front Mol Biosci. 2022 Jan 25;8:791455. doi: 10.3389/fmolb.2021.791455 (PMC8822167; doi:10.3389/fmolb.2021.791455)
Supplement: Supplementary file 1 [file Table2.DOCX]

**Supplementary Table 1**

| **Polysome lysis buffer** (1mL/70 million cells) |  |
| --- | --- |
| Component | final concentration |
| Tris-HCl pH8 | 50mM or 100mM |
| NaCl | 150mM or 30mM |
| MgCl_2_ | 10mM |
| DTT | 1mM |
| IGEPAL | 1% |
| cycloheximide | 100µg/mL |
| Turbo DNase | 24U/mL |
| RNase Inhibitor (RNaseInPlus) | 90U |
| cOmplete Protease Inhibitor (Roche) | 0.33% |
| ddH2O |  |
|  |  |
| **RNaseI footprinting buffer** |  |
| Component | final concentration |
| Tris-HCl pH8 | 100mM or 50mM |
| NaCl | 30mM or 150mM |
| MgCl_2_ | 10mM |
| E-RNaseI (EN0601-Thermo) | 0.8-1U/million cells |
| A-RNaseI (AMM2295- Ambion) | 10-20U/million cells |
| SuperRNase Inhibitor (Ambion) | 3U/million cells |
