# Supplementary material for: Optimization of Ribosome Footprinting Conditions for Ribo-Seq in Human and Drosophila melanogaster Tissue Culture Cells
Source: Front Mol Biosci. 2022 Jan 25;8:791455. doi: 10.3389/fmolb.2021.791455 (PMC8822167; doi:10.3389/fmolb.2021.791455)
Supplement: Supplementary file 2 [file DataSheet1.PDF]

Supplemental Figure 1

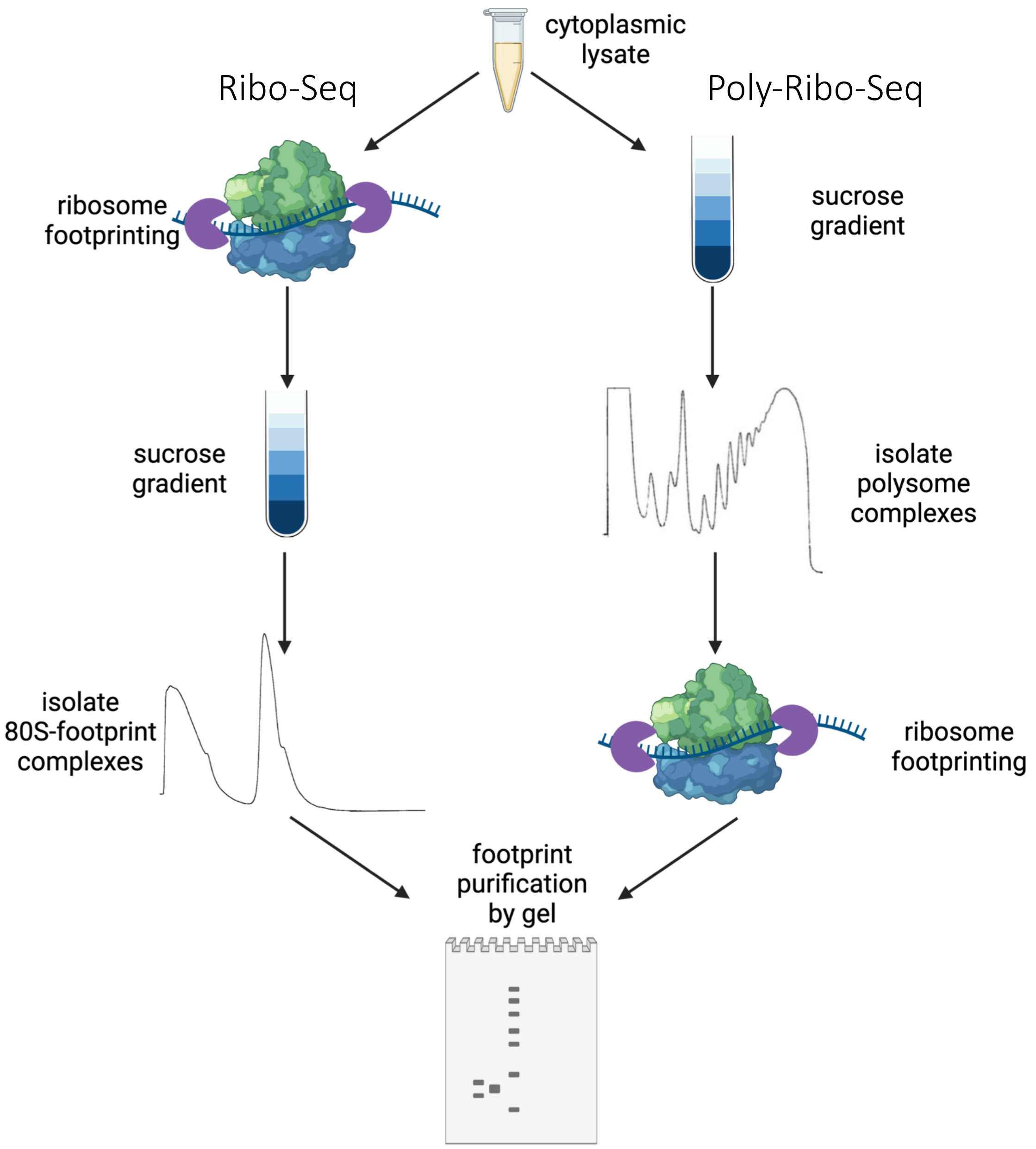

Supplemental Figure 1: Schematic comparison of Ribo-Seq and Poly-Ribo-Seq
In Ribo-Seq ribosome footprinting is performed directly on the cytoplasmic lysate. 80S-footprint complexes are then purified on sucrose cushions. Whilst Poly-Ribo-Seq involves isolating polysomes complexes from cytoplasmic lysate, and then performing ribosome footprinting on this. Both then purify the footprints on urea denaturing acrylamide gels. Figure created with BioRender.com.

Sup 2

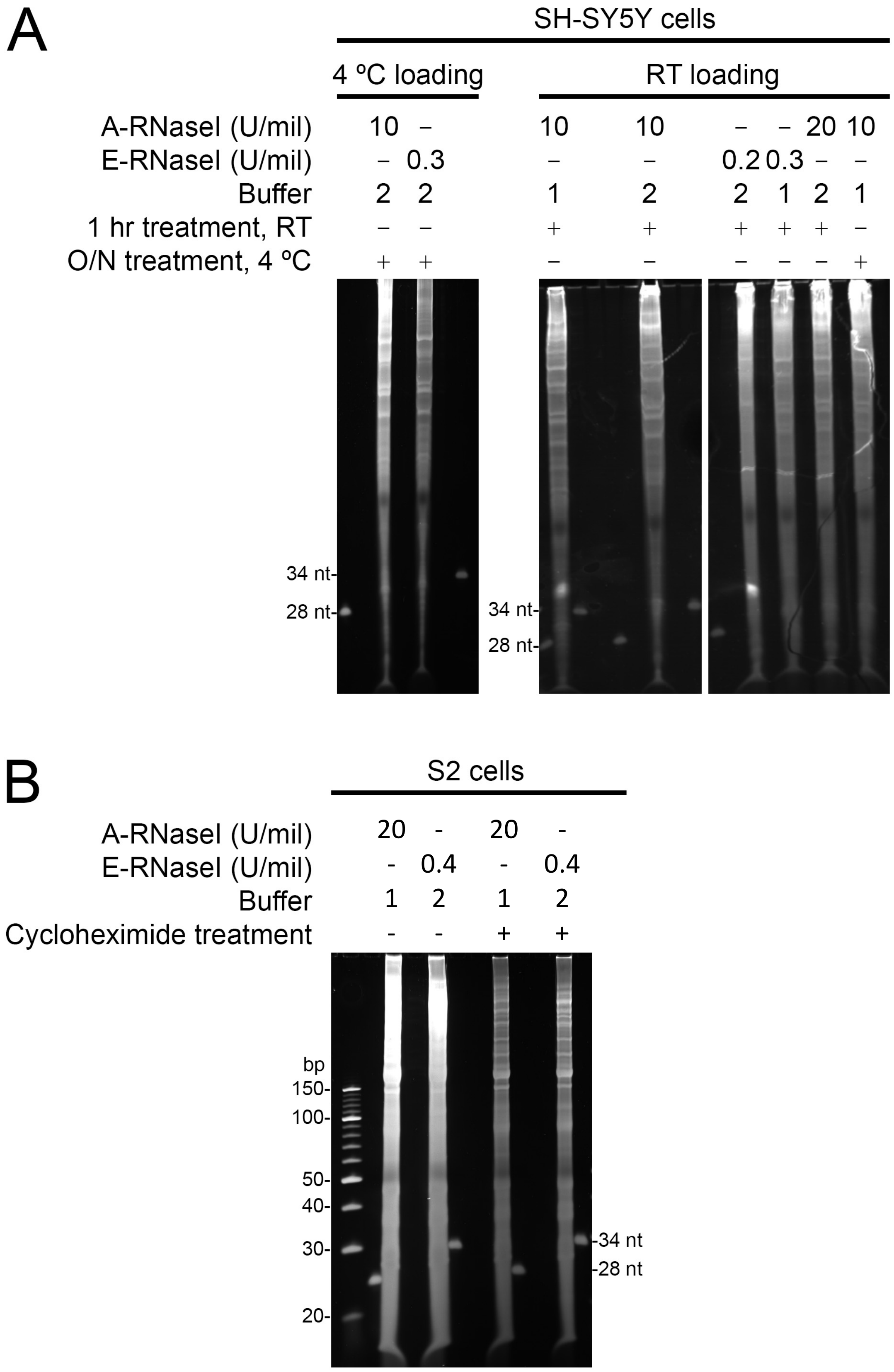

Sup 2: 28–34 nt ribosome footprints are gel purified using 10% (w/v) polyacrylamide-TBE-urea gels
(A) SH-SY5Y samples from the eight different footprinting conditions run on urea-polyacrylamide gels alongside 28 and 34 nt RNA markers to aid with excision. (B) S2 samples from the four different footprinting conditions run on urea-polyacrylamide gels alongside 28 and 34 nt RNA markers, and a DNA ladder. A-RNaseI refers to AM2295, E-RNaseI is EN0601. Buffer 1: 50mM Tris-HCl pH 8 and 150mM NaCl. Buffer 2:100mM Tris-HCl pH 8 and 30mM NaCl.

Sup 3

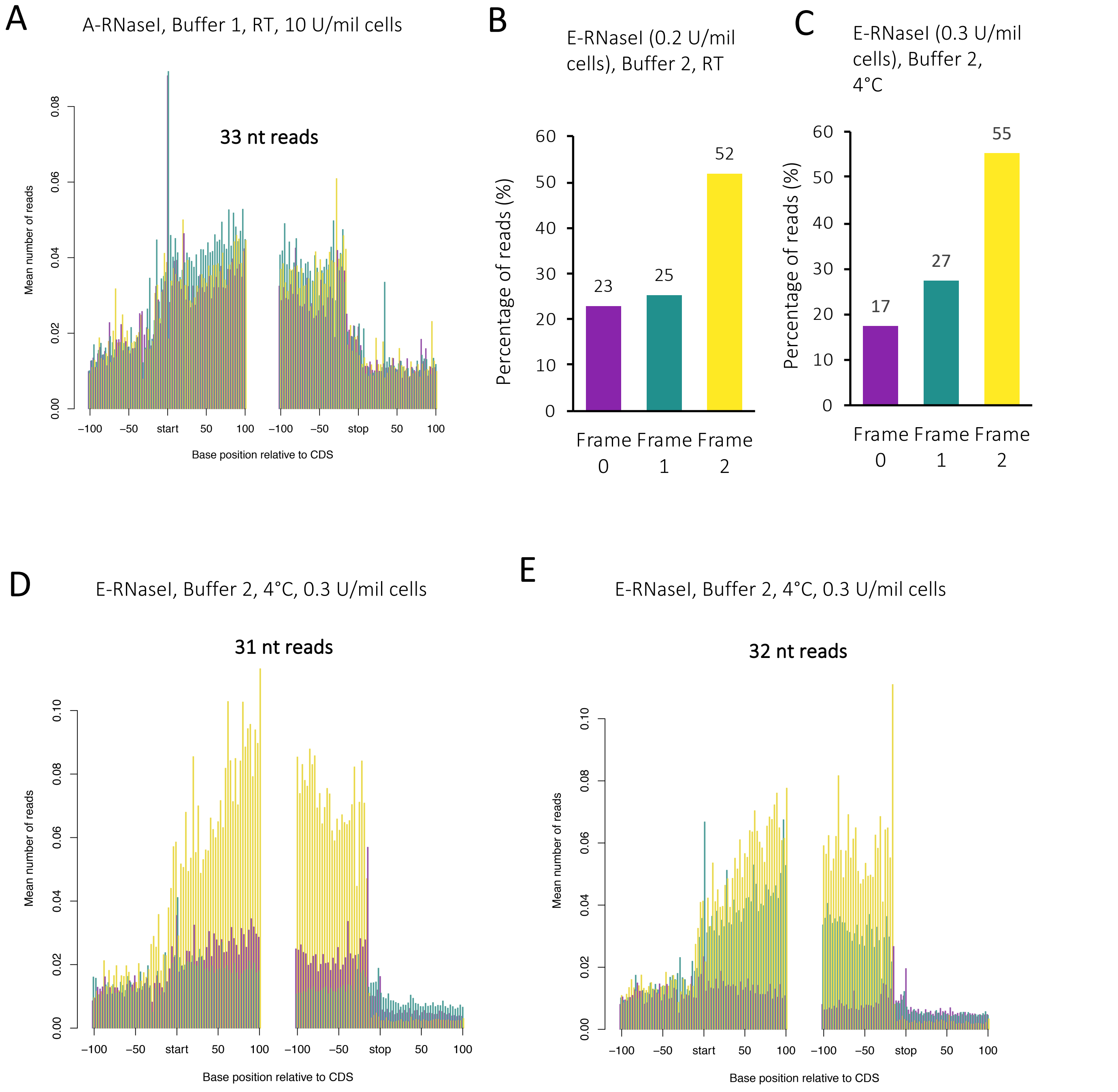

Sup 3: Changes to RNaseI footprinting affects size and framing of ribosome footprints

(A) Metagene analysis in RiboSeqR for 33 nt ribosome footprints from ribosome footprinting performed in SH-SY5Y cells in 50mM Tris-HCl pH8, 150 mM NaCl (Buffer 1), RT 1 hour, 10 U/million with A-RNaseI. (B) Graph of 31 nt reads according to frame for footprints generate in E-RNaseI, 100mM Tris-HCl pH8, 30mM NaCl (Buffer 2), ON at 4, 0.3 U/million cells conditions. Metagene analysis in RiboSeqR from ribosome footprinting performed in E-RNaseI, 100mM Tris-HCl pH8, 30mM NaCl (Buffer 2), ON at 4, 0.3 U/million cells for (D) 31 nt and (E) 32 nt length footprints.

Sup 4

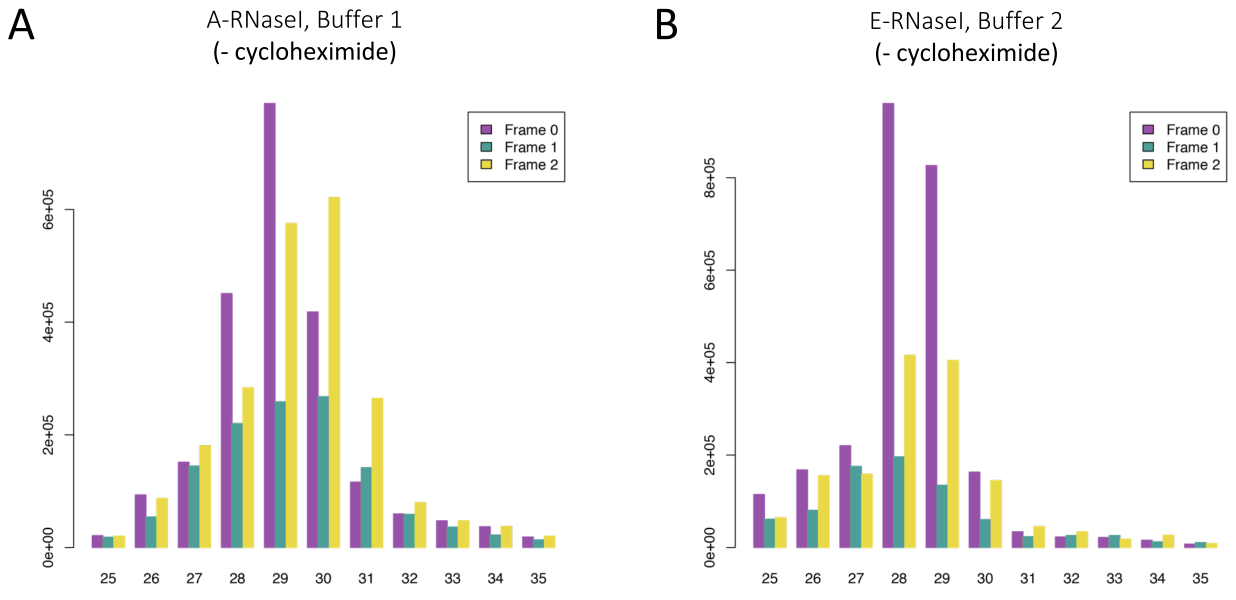

Sup 4: Changes to RNaseI footprinting affects size and framing of ribosome footprints in *Drosophila* S2 cells

Read length distribution and frame plots, generate by RiboSeqR, from cells not treated with cycloheximide, either footprinted in (A) A-RNaseI, Buffer 1 ON at 4°C or (B) E-RNaseI, Buffer 2 ON at 4°.
